# Real-time EEG-based emotion recognition for neurohumanities: perspectives from principal component analysis and tree-based algorithms

**DOI:** 10.3389/fnhum.2024.1319574

**Published:** 2024-03-13

**Authors:** Miguel Alejandro Blanco-Ríos, Milton Osiel Candela-Leal, Cecilia Orozco-Romo, Paulina Remis-Serna, Carol Stefany Vélez-Saboyá, Jorge de Jesús Lozoya-Santos, Manuel Cebral-Loureda, Mauricio Adolfo Ramírez-Moreno

**Affiliations:** ^1^School of Engineering and Sciences, Mechatronics Department, Tecnológico de Monterrey, Monterrey, Mexico; ^2^Fetal-Neonatal Neuroimaging and Developmental Science Center, Boston Children's Hospital, Harvard Medical School, Boston, MA, United States; ^3^School of Humanities and Education, Department of Humanistic Studies, Tecnólogico de Monterrey, Monterrey, Mexico

**Keywords:** EEG, emotion recognition, real-time, PCA, Random Forest, humanities, neurohumanities, Descartes

## Abstract

Within the field of Humanities, there is a recognized need for educational innovation, as there are currently no reported tools available that enable individuals to interact with their environment to create an enhanced learning experience in the humanities (e.g., immersive spaces). This project proposes a solution to address this gap by integrating technology and promoting the development of teaching methodologies in the humanities, specifically by incorporating emotional monitoring during the learning process of humanistic context inside an immersive space. In order to achieve this goal, a real-time emotion recognition EEG-based system was developed to interpret and classify specific emotions. These emotions aligned with the early proposal by Descartes (Passions), including admiration, love, hate, desire, joy, and sadness. This system aims to integrate emotional data into the Neurohumanities Lab interactive platform, creating a comprehensive and immersive learning environment. This work developed a ML, real-time emotion recognition model that provided Valence, Arousal, and Dominance (VAD) estimations every 5 seconds. Using PCA, PSD, RF, and Extra-Trees, the best 8 channels and their respective best band powers were extracted; furthermore, multiple models were evaluated using shift-based data division and cross-validations. After assessing their performance, Extra-Trees achieved a general accuracy of 94%, higher than the reported in the literature (88% accuracy). The proposed model provided real-time predictions of VAD variables and was adapted to classify Descartes' six main passions. However, with the VAD values obtained, more than 15 emotions can be classified (reported in the VAD emotion mapping) and extend the range of this application.

## 1 Introduction

An emotion is a psycho-physiological process triggered by the conscious or unconscious perception of an object or situation. This process is associated with a broad range of feelings, thoughts, and behaviors (Jarymowicz and Bar-Tal, 2006). The study of emotion generation is pivotal, as it underpins the human experience, influencing cognition, perception, and daily activities, including learning, communication, and rational decision-making (Koelstra et al., 2012; Mikhail et al., 2013).

Among the early descriptions of emotion, one is provided by Descartes, which he described as *passions* in his work “The Six Passions of Descartes.” For Descartes, the passions/emotions were experiences of the body on the soul, who, applying his famous method to moral philosophy, represented the problem of the passions of the soul in terms of its simplest integral components, distinguishing six different fundamental passions: admiration, love, hate, desire, joy, and sadness (Descartes, 1649).

According to Descartes, admiration is understood as a sudden surprise of the soul that makes it consider (carefully) objects perceived as rare and extraordinary. This passion is directly related to the search for knowledge and philosophical reflection; love can be interpreted as the origin of the desire for union with someone or something that seems to be convenient; hate leads or drives to the rejection of something or someone; desire leads to an urge of possessing something that is out of reach; joy manifests when someone obtains that which they desire, while under a pleasant situation; lastly, sadness is experienced when losing something desired or during the experience of a painful situation (Descartes, 1649).

The study of emotion has evolved over the years. While early definitions of emotions characterized them as bodily phenomena, modern approaches also acknowledge a cognitive component (Dixon, 2012). The concept of emotion has been delved into both in the Humanistic and Scientific domains.

Carew and Ramaswami (2020) define the humanities as encompassing all facets of human society and culture, including language, literature, philosophy, law, politics, religion, art, history, and social psychology. They underscore the importance of establishing closer collaborations between specific scientific domains, such as Neuroscience and the humanities. They posit that these collaborations will be mutually enriching and usher in a new era of profound and influential academic endeavors (Carew and Ramaswami, 2020). Hartley and Poeppel (2020) contend that advancements in theoretical, computational, neuroimaging, and experimental psychology have enabled linguistics, music, and emotion to emerge as central pillars of contemporary cognitive neuroscience.

In education, the advancement of Humanities teaching methodologies has been notably slower compared to other fields, such as Science and Engineering (Manuel Cebral-Loureda, 2022). This lag underscores the necessity of integrating technology into the Humanities, given its potential to foster human flourishing and enhance emotional education. Recent studies suggest that immersive and interactive teaching environments can significantly improve learning experiences and outcomes (Chih and Lin, 2014). Moreover, the infusion of neuroscience principles into pedagogical strategies is a burgeoning area of interest, with preliminary results indicating promising potential for improved educational experiences (Wilcox et al., 2021).

Developing research-based teaching approaches that combine Neuroscience and Education can help implement immersive and interactive systems for the teaching of Humanities. With this in mind, the *Neurohumanities Lab* project emerges, which intends to implement an immersive and interactive platform for the education of humanities that allows users to interact with the environment (the classroom) and fosters impactful, logical and intuitive learning (Cebral-Loureda and Torres-Huitzil, 2021). The idea behind the Neurohumanities Lab is to integrate a system that can detect movements, actions, emotions, and physiological and mental states through cameras and wearable biometric devices to modify the classroom environment (e.g., through changes in images, lighting, and sound). The proposal for this project is to implement a real-time emotion recognition system using portable Electroencephalography (EEG) that can be integrated into the interactive platform of Neurohumanities Lab.

Our solution is both timely and fitting to address the outlined challenge. It revolves around developing a real-time prediction model for the previously mentioned emotions (admiration, love, hate, desire, joy, and sadness) using brain signals as input. This model is seamlessly integrated into the Neurohumanities Lab's interactive platform. This integration makes the platform an ideal tool for educational innovation, offering students an immersive experience. As they interact within this enriched environment, they can explore and deepen their understanding of Humanities in a classroom setting, which traditionally might not have had such technological engagement. At the same time, students and teachers can understand emotion generation during such experiences and analyze them in context.

Central to our solution is the use of EEG signals. When properly processed, these signals reveal features pivotal for classifying the target emotions. The use of EEG in emotion recognition is not novel; its efficacy has been demonstrated in previous studies (Valenzi et al., 2014; Islam et al., 2021). Many studies suggest that EEG signals provide enough information for detecting human emotions with feature-based classification methods (Valenzi et al., 2014). Others have shown that emotional processing in the brain can be seen from the asymmetry in the brain activity recorded by EEG (Brown et al., 2011).

Various models have been proposed in the intriguing journey to understand and classify human emotions. One of the notable ones is the Circumplex 2D model put forward by Rusell (1980). This innovative model utilizes a two-dimensional approach, mapping emotions based on Valence and Arousal. Valence measures the emotion's intrinsic appeal, determining whether it is perceived as positive or negative. On the other hand, Arousal gauges the level of excitement or intensity associated with the emotion. However, the quest for deeper understanding did not stop there. A subsequent, more intricate, 3D model known as Pleasure, Arousal, and Dominance (PAD), or Valence, Arousal Dominance (VAD), came to the fore (Islam et al., 2021). This model broadened the horizon by incorporating these three elements. While Pleasure and Arousal are reminiscent of Russel's 2D model, adding Dominance provides additional insight. Dominance delves into the realm of control, assessing the extent to which an individual feels in command of, or subdued by, a particular emotion (Islam et al., 2021). This addition elevates the complexity of the model, shedding light on the dynamic interplay between emotions and the sense of power or submission they instill.

The EEG data acquisition process is characterized by several factors: the number of electrodes/channels, electrode placement system on the scalp (measurement of different brain regions), types of stimuli, sampling frequency, and the device used for signal acquisition. The International 10–20 electrode placement system is commonly used for emotion recognition using EEG (Islam et al., 2021).

The most fundamental and challenging task of recognizing human emotion is to find the most relevant features that vary with emotional state changes. The extracted EEG features for shallow and deep learning-based emotion recognition methods are the following: Time-domain features, which include statistical features such as mean, median, standard deviation, mode, variance, minimum, and maximum. The EEG frequency domain features usually contain more relevant information. The main methods are Power Spectral Density (PSD), Fast Fourier Transform (FFT), and the Short Time Fourier Transform (STFT) (Lee et al., 2014; Chaudhary et al., 2016). The Wavelet transform method of analysis presents a good performance both in the time and frequency domain (Mohammadi et al., 2017) and can be classified into two types: Continuous Wavelet Transform (Bostanov and Kotchoubey, 2004) and Discrete Wavelet Transform (Islam et al., 2021). The frequency domain approach was used for this work, focusing on PSD analysis. PSD analysis is a widely used technique for examining the power distribution of various frequency components in a signal and allows us to gain insight into the underlying frequency characteristics of the data. This approach enables the identification of prominent frequency bands or patterns that may indicate specific phenomena or attributes of the EEG signal. In addition, PSD analysis allows for quantifying the relative power contributions of different frequency components, providing valuable information for further analysis and interpretation of the mental states of the participants.

EEG-based emotion recognition systems reported in the literature can be classified into two major groups: Deep machine learning-based and Shallow machine learning-based systems. The first one includes Convolutional Neural Networks (CNN), Deep Neural Networks (DNN), Deep Belief Networks (DBN), Recurrent Neural Networks (RNN), Bimodal Deep Auto Encoder (BDAE), Voting Ensembles (VEn), as classifiers. On the other hand, the second one includes Support Vector Machine (SVM), k Nearest Neighbor (kNN), Random Forest (RF), Decision Tree (DT), Multi-Layer Perceptron (MLP) (Islam et al., 2021). Deep learning techniques are more effective than shallow learning-based algorithms among a wide range of algorithms. However, it may be noted that the SVM performs well in EEG-based emotion. Whenever portability and simplicity are not required, the multimodal data incorporating the other physiological signals (e.g., heart rate, skin conductance, among others) can significantly improve the performance of the emotion recognition system.

In this work, an evaluation of different classification algorithms was implemented to obtain the most accurate model that classifies the desired emotions through VAD estimations in real time. In order to achieve this, a feature extraction, feature selection, and model evaluation process was followed. The length of time windows for the real-time estimation was also included as a parameter when evaluating the models.

Given the aforementioned background, this work describes a real-time emotion recognition algorithm based on VAD estimations for the prediction of the six Descartes' passions. Section 2 presents the details about the dataset used during this work; Section 3 shows a detailed description of the data analysis, feature extraction and selection, and model performance evaluation; Section 4 presents the results of the implementation and a detailed analysis of the variables and models, as well as the specifics of the best predictive model. Finally, Section 5 presents a discussion of the obtained results.

## 2 Materials

### 2.1 Datasets

A review on EEG-based emotion recognition algorithms using deep and shallow learning techniques is presented in Islam et al. (2021), analyzing 41 papers on this topic. Within those articles, 85% use publicly open datasets; in the rest 15%, a self-generated dataset was preferred. Among the 85% articles using publicly available datasets, 61%, 7%, 2%, and 15% of the articles used: A Database for Emotion Analysis using Physiological Signals (DAEP) (Koelstra et al., 2012), The Shanghai Jiao Tong University (SJTU) Emotion EEG Dataset (SEED) (Zheng and Lu, 2015), MAHNOB (Soleymani et al., 2012) and other datasets, respectively. About 26% used the images as stimuli, 23.8% used video, 17.5% used audio, 22.2% used the existing dataset comprising a combination of physiological and emotional data (Alarcão and Fonseca, 2019). The rest of the 10.5% works exploited the emotional data related to games, live performances, or life events. Among these works, different researchers used a diverse range of frequency band-pass filters, and among them, the 4–45 Hz is predominantly used (Lakshmi et al., 2014).

### 2.2 DEAP dataset

One of the main areas where Human-Computer Interfaces (HCI) are deficient is in the field of emotional intelligence. Most HCI systems are unable to interpret information derived from human emotional states and use it to prompt appropriate actions. With this in mind, the article by Koelstra et al. (2012) describes a multimodal dataset that aids in analyzing human affective states. With this objective, the experiment was divided into two parts.

The chosen dataset used to train the model for this project was the DEAP dataset (Koelstra et al., 2012). The first part consists of a self-assessment where 16 subjects observed 120 1-min videos and rated the three variables, Valence, Arousal, and Dominance, on a discrete (1–9) scale. The participants self-rated this scale using the Self Assessment Mannequins (SAM) (Bradley and Lang, 1994). These three planes can be used to quantitatively describe emotion with (1) Arousal, ranging from inactive (uninterested) to active (excited); (2) Dominance, either feeling weak (without control) or empowered (in control); and (3) Valence, ranging from unpleasant to pleasant, where sadness or stress are considered unpleasant and happiness or excitement are considered as pleasant (Koelstra et al., 2012). This dataset also contains an extra value not utilized in the present study: Liking, which measures the extent of positive or negative emotions linked with the given emotional state.

From 120 videos used, 60 were chosen semi-automatically using the “Last.fm” music enthusiast website, which allows users to assign tags to songs and retrieve songs assigned to those tags. In order to select these songs, a list of emotional keywords, as well as inflections and keywords, was used to generate a list of 304 keywords. For each of these keywords, a corresponding tag was searched in the “Last.fm” database, and the ten songs that were most often labeled with this particular tag were selected. This criterion yielded a total of 1,084 songs (from which 60 of them were selected for the experiment). In order to choose these 60 songs, the valence-arousal space was divided into four quadrants, and 15 songs were manually selected for each quadrant. The quadrants are Low Arousal/Low Valence (LALV), Low Arousal/High Valence (LAHV), High Arousal/Low Valence (HALV), and High Arousal/High Valence (HAHV) (Koelstra et al., 2012). In addition to those 60 videos, another 60 were manually selected (15 videos for each quadrant).

For each of the selected (120) videos, a 1-min segment was extracted for the experiment. Subjects were asked to watch each video and provide a VAD rating. Based on the subjective rating obtained from the volunteers, 40 videos were selected out of the original 120 videos, where videos with the strongest ratings and smallest variance were selected for the second part of the experiment (Koelstra et al., 2012).

The second part of the experiment consists of 32 subjects who watched 40 videos in a laboratory environment with controlled illumination and rated through a self-assessment the familiarity of the video on a discrete scale of 1–5 and the liking, arousal, valence, and dominance on a continuous scale of 1–9. While the volunteers were watching the videos, EEG and peripheral physiological signals were recorded, and face video was recorded for 22 participants. Peripheral physiological signals included in this experiment are Galvanic Skin Response (GSR) and Photoplethysmography (PPG).

The database shows an in-depth analysis of the correlates between the EEG signals from the subjects and the subjective ratings given to each video in order to be able to propose a new method for stimuli selection for emotion characterization, providing a statistical analysis of the data obtained (Koelstra et al., 2012).

### 2.3 NHLab functionality

The NeuroHumanities (NH) Lab's immersive platform integrates three primary functionalities: real-time emotion recognition, movement detection, and brain synchronization, which form the foundation of the proposed interactive platform.

The real-time emotion recognition functionality aims to monitor and identify the emotions of individuals within the platform's space. This capability allows for real-time environmental adjustments (colored lights, sounds, music) based on detected emotions and physiological signals, ensuring a tailored experience that aligns with the user's current emotional state. The movement detection functionality provides an interactive dimension to the platform. It enables individuals to interface with the environment using bodily movements and carry out interactive tasks such as painting over a projected screen, selecting words from a projection, and generating changes on images using facial expression recognition. This interaction facilitates active participation and increases user engagement, fostering a more effective learning process. The brain synchronization function seeks to analyze the synchrony between the brain activity of two users. By monitoring the synchronization between EEG channels of both and comparing it with their concurrent behaviors, insights into neural activity related to different aspects of social interactions can be obtained.

By merging these three functionalities, the NH Lab's immersive platform offers a comprehensive educational experience that incorporates real-time emotion monitoring, active user movement interaction, and insights into brain synchronization.

### 2.4 Emotion classification model

**Figure 1:**
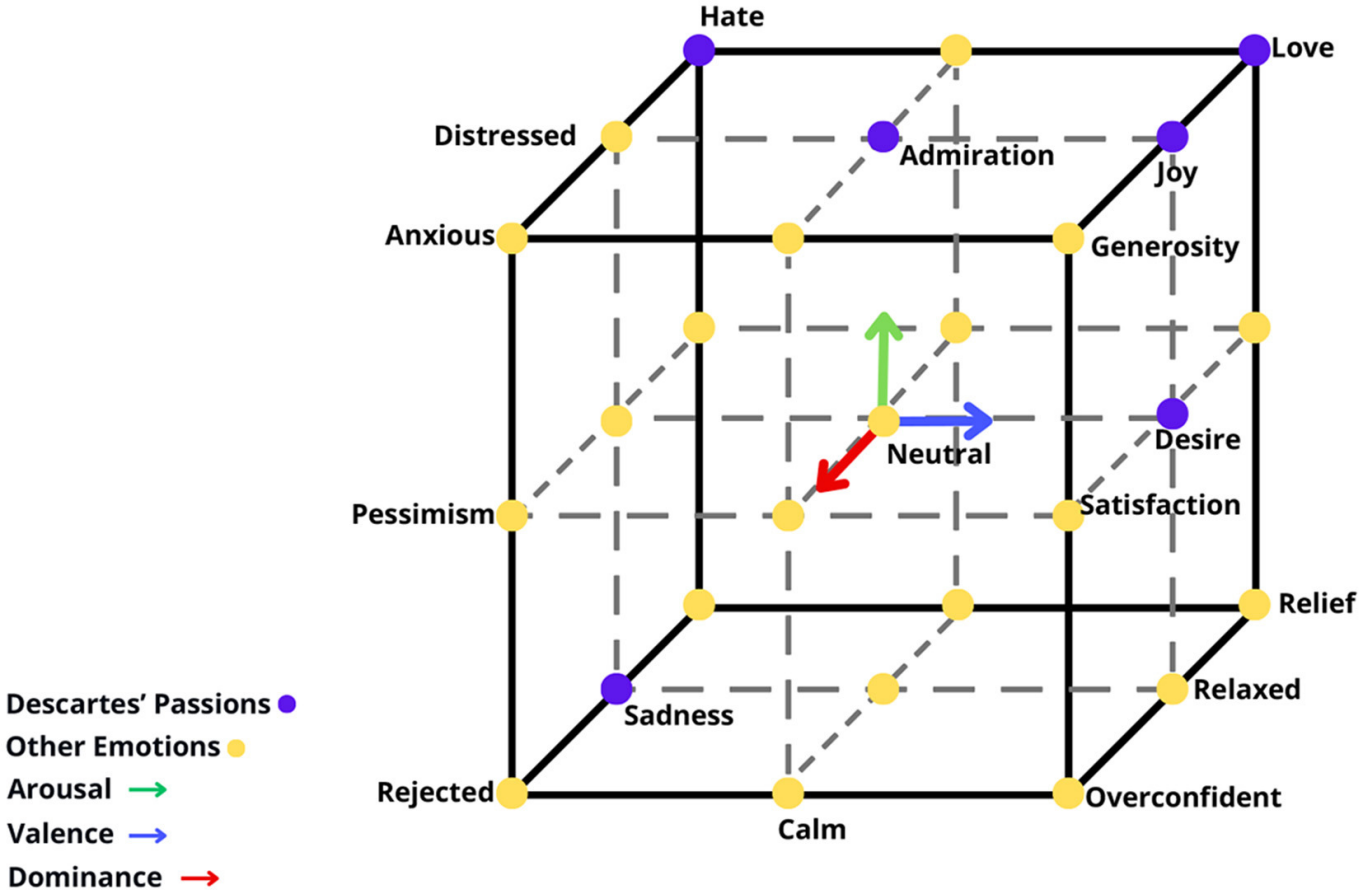
Three-dimensional VAD emotion model based on the work by Islam et al. (2021). Each VAD value had their unique direction and color: Arousal is green and at the *y*-axis (upwards and downwards), Valence is blue and at the *x*-axis (leftwards and rightwards) and dominance is red and at the *z*-axis (in and out). Additionally, Descartes' emotions are colored in purple (Hate, Love, Admiration, Joy, Desire, Sadness), while others are colored in yellow.

The classification model used in this study is based on a 3D model of emotion, which utilizes the VAD framework, shown at Figure 1. This 3D model represents a three-coordinate system, with each coordinate corresponding to one of the VAD labels.

In order to obtain emotions based on the VAD values, a new classification framework was created. Values between 1 and 3.6 were considered low and represented by −1 in the 3D model. Values between 3.7 and 6.3 were considered as medium and represented by 0. Values between 6.4 and 9 were considered high and represented by 1 in the 3D model.

Coordinates were then obtained based on the relationship between the three classes, where each class is associated with a specific emotion. The 3D model of emotion used in this study is based on both (Islam et al., 2021) and the six different passions proposed by Descartes: admiration, love, hate, desire, joy, and sadness. The emotions highlighted in Table 1 are selected for classification in this work. This approach allows for the classification and representation of emotions within the 3D model, providing a framework for understanding and analyzing the participants' emotional responses.

**Table 1:** Relation between the emotion and its coordinate system based on the 3D model at Figure 1.

**Arousal**	**Valence**	**Dominance**	**Emotion**
0	0	0	Neutral
0	0	1	Other
0	0	–1	Other
0	1	0	**Desire**
0	1	1	Other
0	1	–1	Satisfaction
0	–1	0	Other
0	–1	1	Pessimism
0	–1	–1	Other
1	0	0	**Admiration**
1	0	1	Other
1	0	–1	Other
1	1	0	**Joy**
1	1	1	Generosity
1	1	–1	**Love**
1	–1	0	Distressed
1	–1	1	Anxious
1	–1	–1	**Hate**
–1	0	0	Other
–1	0	1	Calm
–1	0	–1	Other
–1	1	0	Relaxed
–1	1	1	Overconfident
–1	1	–1	Relief
–1	-1	0	**Sadness**
–1	–1	1	Rejected
–1	–1	–1	Other

Emotions in bold are Descartes' passions.

## 3 Methods

### 3.1 Data preparation

**Figure 2:**
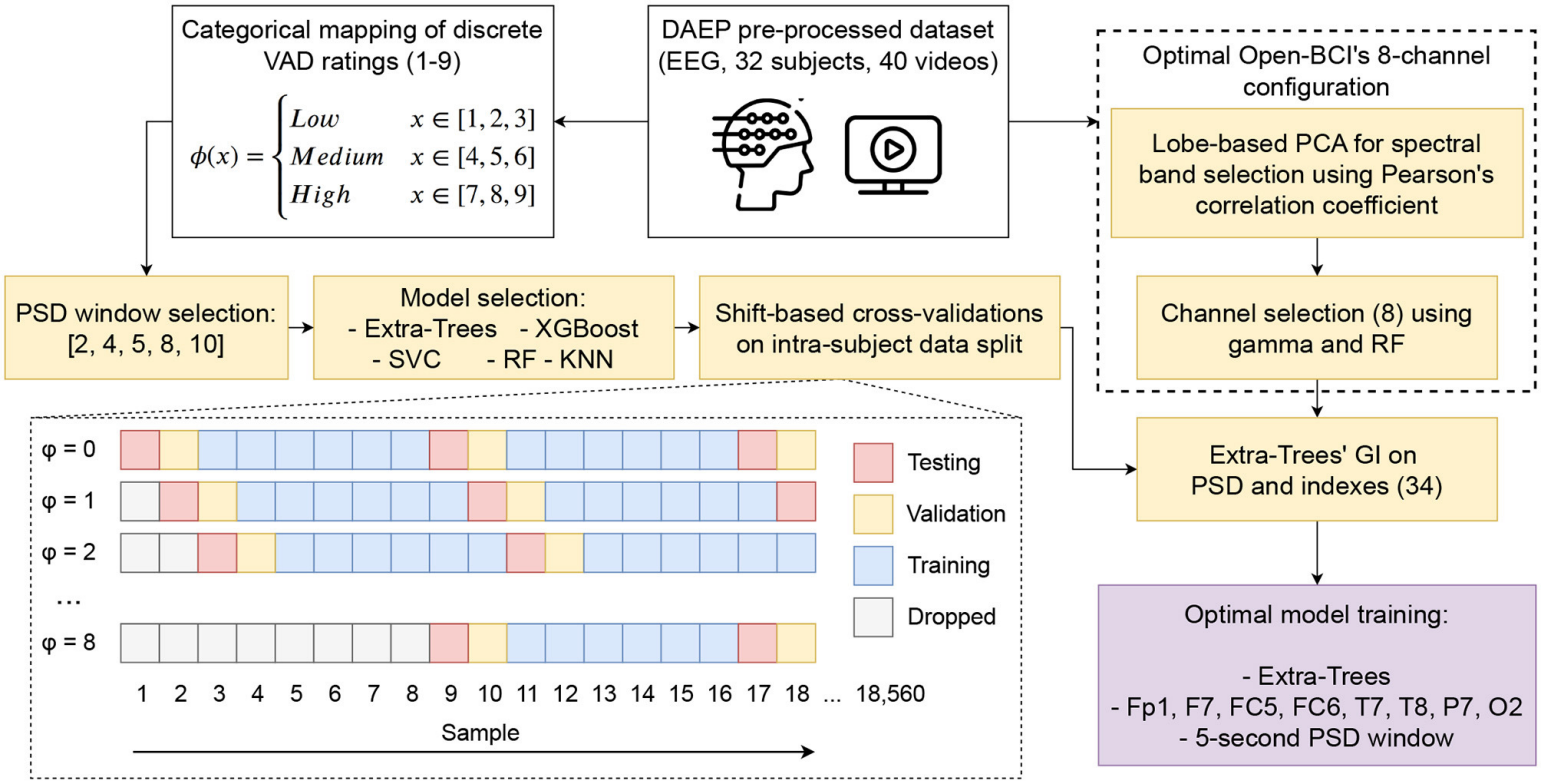
Flowchart of the methodology followed in order to pre-process and process the DAEP dataset to obtain the best-performing model using the optimal hyperparameters (PSD's window length, ML model, 8-channel selection, spectral features, and indexes).

A pre-processed subset of data was used from the aforementioned DEAP Dataset to obtain the desired emotion recognition model. The pre-processing consists of three main steps: (1) downsampling the data from 512 to 128 Hz, (2) applying a band-pass filter between 0.4 and 45 Hz, and (3) averaging the data to the common reference. These files were then combined, and the VAD values were extracted after applying the continuous to discrete transformation explained in Section 2.4. The complete methodological approach can be found in Figure 2, which consists of searching through the best hyperparameters regarding PSD's window length, Machine Learning (ML) model, and helmet's 8-channel configuration. This required a combination of both categorical mapping and continuous VAD ratings, in addition to discriminating according to the model's testing dataset accuracy, validated through a shift-based cross-validation approach regarding the proposed intra-subject data split (60:20:20 when considering training, validation, and testing).

The methodology in Figure 2 was parallel divided into searching for the best parameters; on the left side, using categorical mapping of discrete VAD ratings, the optimal PSD's windows length and ML model was selected using eight-fold shift-based cross-validations on an intra-subject data split framework; while on the right-side finding the eight mostly correlated channels for the OpenBCI helmet with continuous VAD rating through a lobe-based PCA, Pearson's correlation coefficient and RF, thus subsetting from the DEAP's original 32 channels; finally, using the best parameters, spectral features and indexes calculated, on which an optimal 34 features model was fitted. The shift-based cross-validations consist of shifting the index in which the data is partitioned, thus adding a +ϕ to it in order to change the whole distribution of the 18, 560 sample dataset for each subject. The PSD window selection can be found at Section 3.3.1, the model selection at Section 3.5.1, 8-channel configuration at Section 3.4.1, and PSD and indexes at Section 3.3.2.

### 3.2 Preliminary model testing

In the initial stages of the project, a preliminary analysis was performed to obtain a suitable machine-learning model for extracting valuable insights from the EEG data. A comparative study involved three well-established algorithms: the RF Classifier, XGBoost, and the SVM. These models were selected based on their known efficacy in handling high-dimensional data. For the preliminary analysis, data with all the available channels was used to train these models. Evaluation metrics, including accuracy, sensitivity, and specificity, were then used to measure the performance of each model. This foundational step was deemed critical for guiding the subsequent feature and channel selection procedures and validating them.

### 3.3 Feature engineering

#### 3.3.1 PSD estimation

The pre-processed EEG data was segmented into 5-second time windows to capture transient and evolving neural dynamics over time. FFT was then applied to each segment, transforming the raw time-domain EEG data into the frequency domain to obtain PSD using the FFTProcessing function created by Xu (2018).

PSD values were segmented into five distinct frequency bands: Delta (δ) [0.5–4 Hz], theta (θ) [4–8 Hz], alpha (α) [8–12 Hz], beta (β) [12–30 Hz], and gamma (γ) [30–45 Hz].

Selecting an optimal time window length for practical real-time EEG analysis remains critical. A balance between precision and computational speed is essential: while longer windows often provide increased resolution and potential model accuracy, they may compromise processing speed and real-time responsiveness. On the other hand, shorter windows can facilitate rapid processing but might reduce precision. In order to address this balance, the accuracy of VAD prediction was evaluated across different time window lengths and identified an optimal window length. This strategic decision underpins the reliability and efficiency of the real-time implementation.

#### 3.3.2 Frequency band ratios

To aid with the models' performance, different frequency band ratios were implemented for each channel,

*Relaxation Index (*$\frac{\theta }{\delta }$*)*: this ratio accentuates the interplay between the theta waves, linked with drowsiness or light meditation states, and delta waves, associated with deep sleep and unconscious processes (Machado et al., 2010). An elevated Theta/Delta ratio often suggests a state of relaxation without lapsing into deep unconsciousness, making it a valuable marker for assessing relaxation in awake individuals.

*Excitement Index (*$\frac{\beta }{\alpha }$*)*: this ratio is served as an indicator of attention and engagement. It is suggested by a higher ratio that individuals are more alert and attentive, which can be interpreted as excitement or heightened interest. In the context of the research paper, the efficiency of advertisements at a population level was predicted using this ratio, indicating that ads evoking higher engagement, as measured by the beta/alpha ratio, are deemed more effective (Kislov et al., 2023).

*Mental Fatigue Index (*$\frac{\alpha }{\theta }$*)*: by examining the Alpha/Theta ratio, signs of mental weariness can be observed. A predominant alpha wave activity in relation to theta can signify a relaxed or idling state of the brain, which, especially during tasks requiring sustained attention, can indicate cognitive fatigue (Ramírez-Moreno et al., 2021).

*Engagement Index (*$\frac{\beta }{\theta +\alpha }$*)*: in order to represent the balance of active cognitive processing vs. a more passive state, this ratio is particularly crucial in contexts where the depth of cognitive immersion or focus is under scrutiny. A high value suggests robust cognitive engagement or alertness, paramount in activities demanding continuous mental effort (Ismail and Karwowski, 2020).

### 3.4 Feature selection

#### 3.4.1 Channel selection using PCA and RF

Among the crucial features of the real-time model, it is noteworthy that the evaluated dataset includes data from 32 EEG channels; however, the proposed real-time algorithm is intended to be integrated into an 8-channel, dry-electrode OpenBCI system (OpenBCI, New York, NY, USA) for a highly-portable, practical implementation (Lakhan et al., 2019; Zhou et al., 2019). Many studies have used only 8 channels to obtain EEG signals (Brown et al., 2011; Mikhail et al., 2013; Valenzi et al., 2014; Zhou et al., 2019). This 8-channel system allows the re-configuring of different positions of electrodes around the scalp.

The channel selection was conducted to assess which are the best 8 channels to use by the OpenBCI for the proposed emotion recognition algorithm. Following this idea, channels were grouped into their respective lobes and joined their data via dimensionality reduction techniques; then, three RF models were fitted (one for each emotion component) in order to assess lobe importance with respect to a series of frequency bands.

Feature importance becomes quite challenging given the high dimensionality of source features (32 channels · 5 frequency bands = 160), and the vast amount of total samples (593, 920; 58 seconds of a video clip through a 0.125 seconds moving window, 58/0.125 = 464 samples for each 40 videos and 32 subjects, 464·40·32 = 593, 920). Regression models such as RF would be not only slow due to high dimensionality, but slight differentiation between features' importance from one to another would be absent due to using normalized relative importance, which gives each feature an importance such that the sum of importance is equal to 1.

PCA was used to reduce the dimensionality of the data; it was applied to source features regarding their channel anatomical location, separated within the Frontal, Temporal, Parietal, Occipital, Central, and Central-Parietal lobes, for each frequency band, and subjects' data. Table 2 shows which EEG channels were assigned to which lobe.

**Table 2:** Brain lobes and channels are assigned to each lobe regarding brain anatomy; although some lobes have more channels than others, PCA always reduced the dimensionality to *k* components.

**Lobe**	**Channels**
Frontal	Fp1, Fp2, F3, F4, F7, F8, Fz, AF3, AF4
Temporal	T7, T8
Parietal	P3, P4, P7, P8, Pz, PO3, PO4
Occipital	O1, O2, Oz
Central	FC5, FC1, C3, C4, FC2, FC6, Cz
Central-Parietal	CP5, CP1, CP2, CP6

The objective of applying PCA to the dataset is to gather insights about the brain region whose features are most linearly correlated to the target features, which would suggest a strong relation. The calculation of the PCA first consists of the Singular Value Decomposition (SVD) technique, which decomposes matrix *X* into the matrix multiplication of three matrices *U* Σ *V*^*T*^, as in Equation 1.


(1) $$\begin{array}{l} X=U\cdot \Sigma \cdot V^{T} \end{array}$$

*V* has the unit vectors of the *k* components, represented in Equation 2.


(2) $$\begin{array}{l} V=\left(\begin{array}{cccc} | & | & |\\ c_{1} & c_{2} & \cdots & c_{n}\\ | & | & | \end{array}\right) \end{array}$$

*V* has *n* × *k* dimensionality, although we only require the first component in order to compress all the channels' vectors on each lobe to a single vector. So for each *V* matrix calculated, when considering the first component *V*_1_, for each lobe *l* and frequency band *b* with respect to a subject *s*, the whole *X* data (consisting of *n* samples by *m* channels), was transformed to a *Z* vector (consisting of *n* samples by 1 dimension), thus leaving a unique vector of values for each combination of *l* and *b* on a subject *s*. The computation can be seen in Equation 3.


(3) $$\begin{array}{l} Z_{s,l,b}=X_{s,l,b}\cdot V_{1,s,l,b} \end{array}$$

Pearson correlation coefficient is calculated for each subject *s* as in Equation 4; afterwards, an average across *S* subjects was calculated; variance between each subject's data was thus reduced as if PCA would be calculated on all complete data, features' domain between subjects would make PCA unstable, as the technique assumes that the dataset is centered around the origin; where *z*_*i*_ represents each sample for each PCA *l* lobe and *b* frequency band, while *y*_*i*_ represents each sample for each *e* emotion VAD component. Thus, a single mean absolute correlation coefficient is calculated for each source feature, averaged among each subject's data. This coefficient was further used in order to determine which channels are the most linearly correlated to the target emotion VAD, hence reducing the dimensionality of the whole dataset when sub-setting the best lobes on a particular frequency band for each emotion component.


(4) $$\begin{array}{l} r\left(l,b,e\right)=\frac{1}{S}\overset{S}{\underset{s=1}{\sum }}|\frac{\Sigma \left(z_{i,s,l,b}-{\bar{z}}_{s,l,b}\right)\left(y_{i,s,e}-{\bar{y}}_{s,e}\right)}{\sqrt{\Sigma {\left(z_{i,s,l,b}-{\bar{z}}_{s,l,b}\right)}^{2}\Sigma {\left(y_{i,s,e}-{\bar{y}}_{s,e}\right)}^{2}}}| \end{array}$$

Once the most relevant lobes were found, an RF regressor was used to determine feature importance, thus obtaining the best 8 channels using feature selection. The RF model from Sklearn, a ML package from Python, contains the Gini importance (GI) metric, which is the normalized total reduction of a given criterion brought by each feature. On classification, it represents the number of splits in a decision tree that used that feature within the RF ensemble (Morales-Menendez et al., 2021); while on regression, it uses the Mean Squared Error (MSE) criterion, which follows the Euclidean norm, thus reducing the Euclidean distance between two points in a given vector space (Candela-Leal et al., 2022) and giving highest importance to the feature that reduced most variance.

The MSE criterion is shown in Equation 5, which is essential when calculating GI in RF regression; the reduction of variance is calculated by minimizing the squared difference between target feature *y* and predicted target feature ŷ, based on the RF model. The channel *c*, whose inclusion as a feature leads to the greatest reduction of this criterion, will also exhibit the highest GI, normalized across all channels *C*.


(5) $$\begin{array}{l} MSE\left(c,e\right)=\frac{1}{N}\overset{N}{\underset{i=1}{\sum }}{\left(y_{i,e}-{ŷ}_{i,c,e}\right)}^{2} \end{array}$$

GI was obtained for each subject's data *s* and then averaged across the total number of subjects *S*. Since RF is randomly initialized, *I* iterations were carried out to generalize better and ensure that a random initialization would not benefit a specific feature (in which *I* = 10). Therefore, for each subject *s*, *I* RF models with different random initializations were run, and GI was averaged to obtain GI for a specific subject. Furthermore, each subject's GIs were averaged to obtain a global importance based on all subjects. This aforementioned calculation is shown in Equation 6.


(6) $$\begin{array}{l} GI\left(c,e\right)=\left(\frac{1}{S}\right)\left(\frac{1}{I}\right)\overset{S}{\underset{s=1}{\sum }}\overset{I}{\underset{i=1}{\sum }}GI{\left(c,e\right)}_{i,s} \end{array}$$

Considering only the best band and lobe combination, the criterion is computed for *C* channels (*C* = 32); hence, the normalized feature selection criterion would be capable of detecting slight importance changes between features. Three RF models, one for each emotion *e* component VAD, would be fitted; thus, each emotion component would have their best set of features, in which Equation 7 must be satisfied.


(7) $$\begin{array}{l} \overset{C}{\underset{c=1}{\sum }}GI\left(c,e\right)=1 \end{array}$$

Given that the three GI would have the same domain and the same number of features, an Emotion Importance Index (EII) was calculated in order to evaluate the overall importance of each feature to the process of predicting *E* emotions, as in Equation 8, hence proposing a more holistic approach on the feature selection process of selecting the best 8 EEG channels.


(8) $$\begin{array}{l} EII\left(c\right)=\frac{1}{E}\overset{E}{\underset{e=1}{\sum }}GI\left(c,e\right) \end{array}$$

### 3.5 Model evaluation

#### 3.5.1 Model selection

A range of metrics was adopted to identify the optimal classifier model. Recognizing that a single metric might not fully capture a model's effectiveness, especially with varied data distributions, two metrics were employed: accuracy and F1-Score. These metrics were selected to offer a comprehensive understanding of model performance and to ensure a reliable choice was made. Metrics were calculated as in Aguilar-Herrera et al. (2021), where True Positives (TP), True Negatives (TN), False Positives (FP) or type-I errors, and False Negatives (FN) or type-II errors are used to build up these metrics.

*Accuracy:* In the model selection process, accuracy was utilized as a primary metric. Defined by the equation:


(9) $$\begin{array}{l} \text{Accuracy}=\frac{TP+TN}{TP+TN+FP+FN} \end{array}$$

Equation 9 represents the ratio of correctly predicted observations to the total observations. This measure assessed the overall correctness of the model's predictions.

**F1-Score:** Given the potential pitfalls of using accuracy alone, especially in imbalanced datasets, F1-score was also employed for further depth in evaluation (Equation 10). The metric represents the harmonic mean of the positive predictive value, or precision (Equation 11) and the true positive rate, or recall (Equation 12).


(10) $$\begin{array}{l} \text{F1-Score}=2*\frac{\text{Precision}\cdot \text{Recall}}{\text{Precision}+\text{Recall}} \end{array}$$

Where


(11) $$\begin{array}{l} \text{Precision}=\frac{TP}{TP+FP} \end{array}$$

And


(12) $$\begin{array}{l} \text{Recall}=\frac{TP}{TP+FN} \end{array}$$

This metric gives a more nuanced understanding of the model's performance on both the positive and negative classes.

By employing these metrics, a comprehensive understanding of the performance of different classifier models was ensured, allowing for a more informed model selection to be made. For each of the VAD classes (Low, Medium, and High), a total of five different classifier models were trained. Including three tree-based classification models: Extra-Trees (ET), Random Forest (RF), XGBoost (XGB); as well as other models such as k-Nearest Neighbor (kNN), Support Vector Classifier (SVC).

#### 3.5.2 Feature importance selection

Once the model was selected, its performance was evaluated over a range of feature subsets, specifically between 20 and 40 features. The optimal subset was identified based on the best performance metrics. The feature importance function from scikit-learn, applied using the ExtraTrees model, was utilized for this assessment.

In tree-based models such as Extra-Trees, a feature's importance is determined by the frequency and depth of its use for data splits across all trees (Olivas et al., 2021). A feature frequently used and closer to the tree roots is considered more crucial. The importance of a feature is typically calculated by averaging the decrease in impurity, often quantified using the Gini criterion, across all nodes where the feature facilitates a split (Martínez et al., 2021). By aggregating over the ensemble of trees, more robust and less biased feature importances are typically achieved.

### 3.6 Real-time implementation

In this work's final phase, the real-time application was developed. Emotion recognition was achieved by integrating insights derived from three distinct ML models. This methodology was further enriched by including the three-dimensional emotion model proposed by Islam et al. (2021).

**Figure 3:**
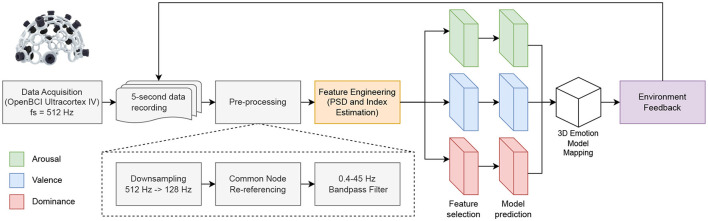
Flowchart of the real-time emotion recognition implementation.

Figure 3 shows the real-time pipeline implemented for the EEG-based VAD estimation and thus detecting of Descartes' passions.

The pipeline consists of retrieving EEG signals from an 8-channel OpenBCI Ultracortex IV EEG helmet using a Cyton board. Further, using 5-second data and pre-processing it according to Section 3.1, features are created according to Section 3.3, further subsetting the best features according to each VAD model, as well as their respective prediction. Finally, the user received feedback based on the VAD predictions and the 3D emotion model mapping described in Figure 1. The OpenBCI Ultracortex IV EEG helmet has a total of 35 possible node locations, with the default version being FP1, FP2, C3, C4, P7, P8, O1, and O2. However, these channels were used in the initial iteration of the project; these would be further replaced with the optimal emotion recognition channels regarding the channel selection results in Section 4.2.

**Figure 4:**
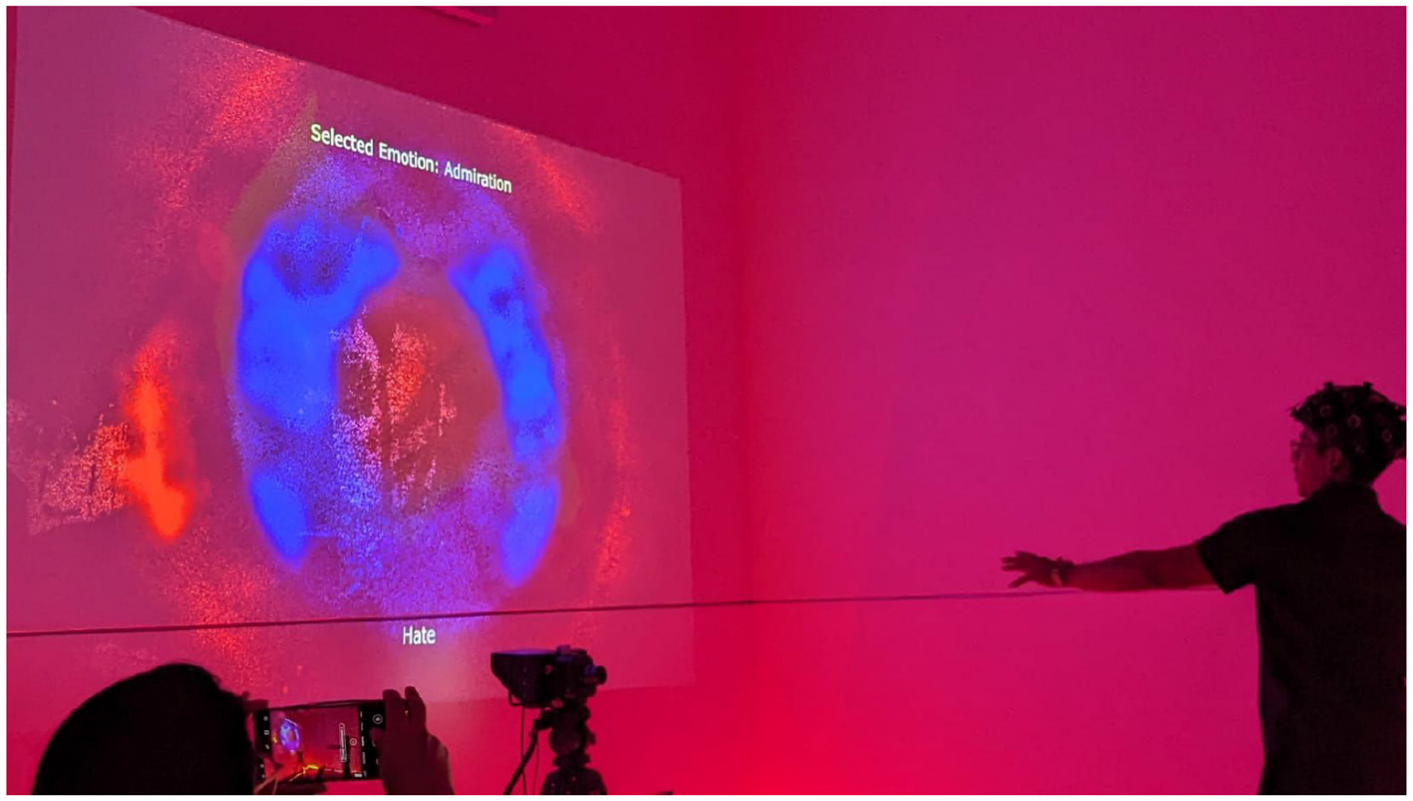
Testing the real-time application of the algorithm at the NeuroHumanities Lab at Tecnologico de Monterrey, Campus Monterrey.

On the other hand, the real-time use of the NH Lab platform can be seen in Figure 4. In this platform, the user wears the OpenBCI helmet, and the emotion recognition model identifies in real-time one of the six Descartes' passions. Depending on the detected emotion, the lighting of the environment changes. A camera, and a motion tracking algorithm are used to detect the users' movements that allow the generation of a painting on the projected screen. The color palette of the visualization, as well as sound effects and music related to the movement, are different depending on the detected emotion.

Moreover, the participants wear an Empatica E4 (Empatica Inc, Milano, IT), which measures Electrodermal Activity (EDA), Blood Volume Pulse (BVP), three-axis acceleration, and temperature. Empatica E4 signals are also acquired in real time. Changes in these values are reflected in the system as well; for instance, the volume of specific sounds related to the EDA (electrostatic noise) and BVP (heartbeats) changes in accordance with increases/decreases of the estimated engagement (via EEG).

## 4 Results and discussion

### 4.1 Time window selection

**Figure 5:**
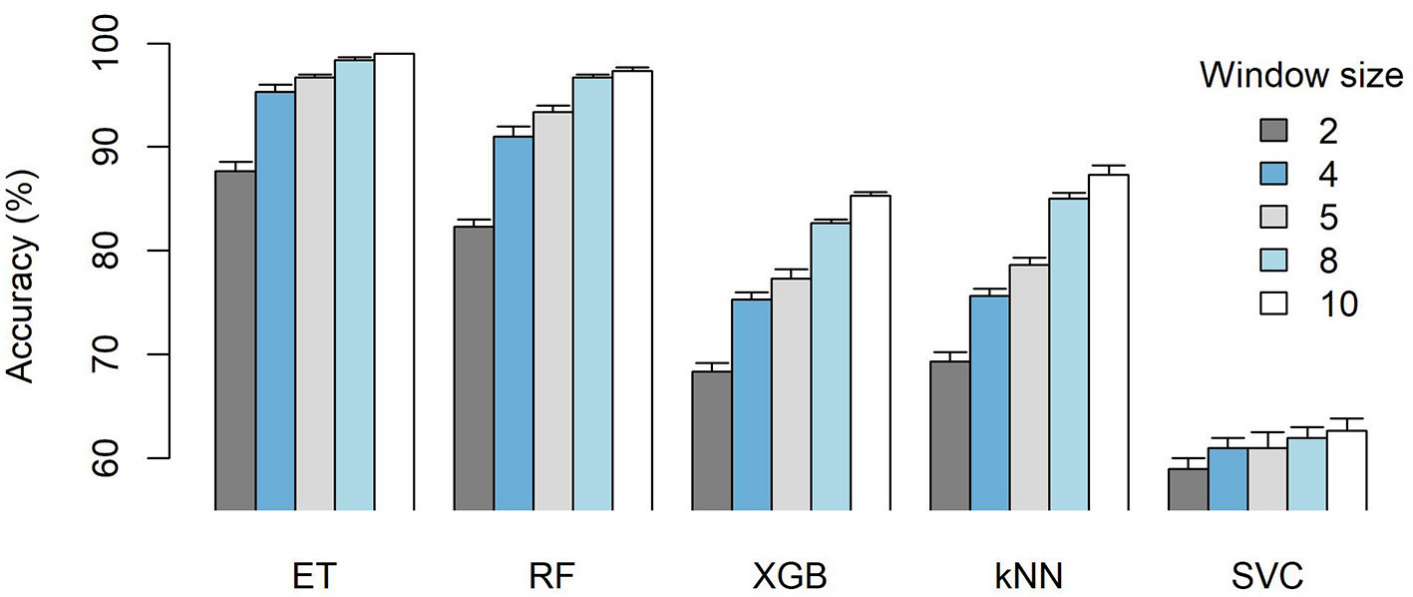
Accuracy comparison of different time windows (2, 4, 5, 8, 10 seconds), prior to the feature selection process, for each ML model averaged across the three VAD components.

Using the standard OpenBCI channel configuration, PSD was obtained over various time window lengths, ranging from 1 to 10 seconds. In order to determine the optimal window length, the performance-to-time ratio was considered, especially since the intended application was in real-time scenarios (Lozoya-Santos et al., 2022). The results for time windows of 2, 4, 5, 8, and 10 seconds are presented in Figure 5.

Based on the results, different time windows have different behaviors on each of the ML models. SVC has the lowest performance, and the higher window size does not improve its accuracy significantly. On the other hand, both XGB and kNN have a steeper increase in accuracy according to the window size length. Finally, both ET and RF exhibit similar behavior, as a steep increase in accuracy is shown between 2- and 4-second windows and follows a gentler increase in accuracy between 5-, 8-, and 10-second windows, thus following an asymptotic behavior as it approaches perfect accuracy. From the results, the 5-second time window is promising for the final model since it provided an average accuracy on the top-performing models of 96% at ET and 93% at RF, in addition to having no significant improvements for the 8-second (98% ET, 96% RF) and 10-second 99% ET, 97% RF) windows. Therefore, given the positive accuracy and the fast implementation it entails, the 5-second window was selected.

### 4.2 Channel selection

According to the lobe regions defined in Table 2, Sklearn library from Python was used to obtain the first component for each lobe and spectral band combination (6 lobes · 5 spectral bands = 30). This process significantly reduces the number of features analyzed, in contrast to the initial (32 channels · 5 spectral bands = 160). After the PCA was calculated for each subject, the mean absolute Pearson correlation coefficient was obtained, as stated in Equation 4, which provided a unique importance value for each combination tested.

Results of the PCA-found features' correlation are shown in Table 3. A unique VAD value was calculated for each frequency b and lobe combination; the summation of the three coefficients was also calculated to evaluate overall feature importance. The highest linearly correlated features correspond to the γ frequency band, as it had the best overall correlation among VAD for each lobe: Frontal with 0.4272, Temporal with 0.4433, Parietal with 0.4585, Occipital with 0.4563, Central with 0.4443, Central-Parietal with 0.4251; along with the best correlation coefficients for each emotion component: γ_*O*_ for Arousal with 0.2011, γ_*P*_ for Valence with 0.1316, γ_*T*_ for Dominance with 0.1327.

**Table 3:** Mean absolute Pearson correlation coefficients between the emotion component and each lobe's first Principal Component across each subject.

***f*_*band*_**	**Lobe**	**Arousal**	**Valence**	**Dominance**	**Σ**
δ	F	0.0472	0.0447	0.0422	0.1341
	T	0.0511	0.0455	0.0436	0.1402
	P	0.05	0.0435	0.0445	0.138
	O	0.0438	0.0423	0.0426	0.1287
	C	0.0511	0.043	0.0447	0.1388
	CP	0.0456	0.0446	0.0406	0.1308
θ	F	0.0877	0.0795	0.0778	0.245
	T	0.0954	0.0804	0.0785	0.2543
	P	0.0971	0.083	0.0833	0.2634
	O	0.0946	0.0762	0.0794	0.2502
	C	0.0945	0.0783	0.0812	0.254
	CP	0.0839	0.083	0.0773	0.2442
α	F	0.1146^*^	0.0686	0.0763	0.2595
	T	0.1123	0.073	0.078	0.2633
	P	0.1138	0.087	0.0845	0.2853
	O	0.1233	0.0883^*^	0.0844	0.296
	C	0.1182^*^	0.0828^*^	0.0836	0.2846
	CP	0.1108	0.0815	0.08	0.2723
β	F	0.1642	0.0989	0.1065	0.3696
	T	0.1684	0.0988	0.1183	0.3855
	P	0.1894	0.1113	0.1184	0.4191
	O	0.1797	0.1072	0.1094	0.3963
	C	0.1788^*^	0.0985	0.1207	0.398
	CP	0.1696^*^	0.1083	0.1084	0.3863
γ	F	0.1868^*^	0.1228	0.1176^*^	**0.4272**
	T	0.188^*^	0.1226^*^	**0.1327**	**0.4433**
	P	0.2005	**0.1316** ^*^	0.1264	**0.4585**
	O	**0.2011**	0.1295	0.1257	**0.4563**
	C	0.1958	0.1178	0.1307	**0.4443**
	CP	0.1783^*^	0.1281	0.1187	**0.4251**

^*^p < 0.05 for >95% of the subjects. F, frontal; T, temporal; P, parietal; O, occipital; C, central; CP, central-parietal.

The highest values for Arousal, Valence, and Dominance are marked in bold, in addition to the highest sum of VAD coefficients for each lobe. Statistically significant linearly correlated for more than 95% of the subjects (31 out of 32 or 32 out of 32 subjects) are marked with a ^*^.

There is a linear correlation between frequency bands and the sum of Pearson correlation coefficients; when frequency increases, this coefficient also increases. Considering the overall sum coefficients for each lobe, on each frequency band: δ has coefficients between 0.12 and 0.14, θ has coefficients between 0.24 and 0.26, α has coefficients between 0.25 and 0.29, β has coefficients between 0.36 and 0.41, γ has coefficients between 0.42 and 0.45. Hence, based on those overall coefficients, there seem to be three clusters: δ, θ, and α, β and γ, with the lowest frequency bands being the least linearly correlated and the highest frequency bands being the most linearly correlated. These results are similar to the reported by other authors (Li and Lu, 2009; Martini et al., 2012; Yang et al., 2020), who also determined that γ bandpower in EEG is the most suitable for emotion classification.

It is important to note that linear correlation does not directly mean more feature importance, as some features might be non-linearly correlated and still be critical features for the target feature prediction. However, for the first assessment and feature discrimination, the assumption of higher lineal correlation means higher feature importance is made. Furthermore, an RF regression model would be fitted on the best frequency band's channels in order to gather true feature importance with respect to the GI criterion, which does not necessarily give importance to linearly correlated features with the target emotion.

Regarding statistically significant linearly correlated features for more than 95% of the subjects on at least one of the VAD emotion components, the higher frequency bands trend is displayed, as 4 features correspond to the γ band, 2 to the β band, and 3 to the α band. Arousal and Valence have several statistically significant linearly correlated features (α_*F*_, α_*C*_, β_*C*_, β_*PC*_, γ_*F*_, γ_*T*_, γ_*PC*_) and (α_*O*_, α_*C*_, γ_*T*_, γ_*P*_) respectively, although Dominance only has γ_*F*_.

**Figure 6:**
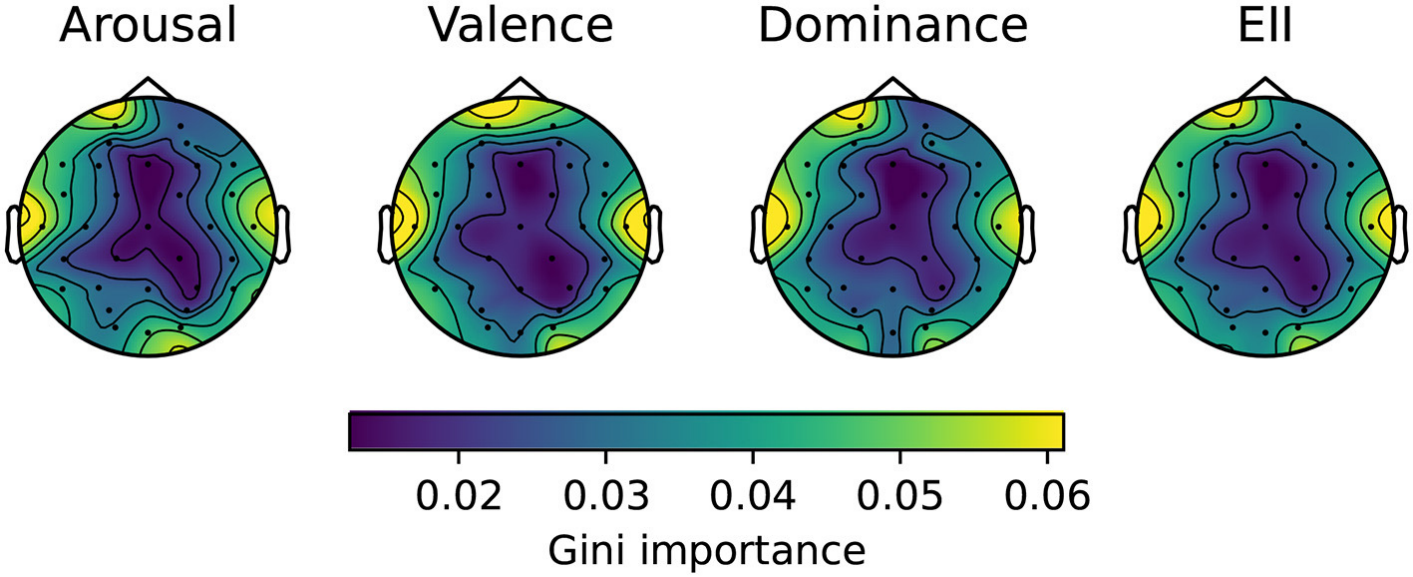
Average GI topoplot for each channel, considering only γ frequency band (30–45 Hz) and fitting a separate RF model on Arousal, Valence, and Dominance. EII was calculated by averaging GIs across emotion components as described in Equation 8.

In order to assess which are the best 8 channels to use in the OpenBCI helmet (available for real-time implementation), the best 6 lobes with the highest sum of VAD coefficients were considered (γ_*F*_, γ_*T*_, γ_*P*_, γ_*O*_, γ_*C*_, γ_*PC*_). Hence, all the lobes on the γ frequency band would be a good choice, so each channel *c* would have an assigned importance value (higher is better), calculated based on Equation 6, described in Section 3.4.1. In order to visually understand the importance of each channel, a topoplot was created, illustrating a spatial map of the obtained GI values. The topoplot is shown in Figure 6.

Overall, Arousal, Valence, and Dominance topoplots are similar, as the channels at the center of the brain are not as important as the ones at the exterior part of the brain; this pattern is also shared on EII, which displays the average patterns found on each of other three emotion component's plot. Focusing on EII's topoplot better generalizes overall feature importance on determining which channel is more related to VAD values, and thus it is more useful when identifying emotions.

The coefficients are also presented in Table 4. Both T7 and T8 have the highest GI importance according to EII (0.0586, 0.0557), which are the only channels from the temporal lobe, following Fp1 (0.0519), and F7 (0.0442) from the frontal lobe. These results are similar to the reported by Zhang et al. (2016) and Wang et al. (2019), where temporal and frontal regions had the highest importance for emotion recognition.

**Table 4:** GI for each 32 EEG DAEP channel at their γ bandpower, ordered from highest to lowest importance according to EII.

**Channel**	**A**	**V**	**D**	**EII**	**Channel**	**A**	**V**	**D**	**EII**
T7	0.0561	0.061	0.0586	0.0586	C4	0.0284	0.0302	0.0289	0.0292
T8	0.0527	0.0611	0.0534	0.0557	CP6	0.0292	0.0271	0.0299	0.0287
Fp1	0.0495	0.0523	0.0539	0.0519	P3	0.0292	0.0301	0.0268	0.0287
F7	0.0439	0.0435	0.0451	0.0442	CP5	0.0248	0.0294	0.0295	0.0279
FC5	0.0425	0.0433	0.0444	0.0434	PO3	0.0291	0.027	0.0256	0.0272
O2	0.0442	0.0429	0.0426	0.0432	C3	0.0311	0.022	0.0271	0.0267
P7	0.0374	0.0419	0.0371	0.0388	FC1	0.0242	0.0239	0.0283	0.0255
FC6	0.0439	0.0364	0.0359	0.0387	Pz	0.0273	0.0195	0.0265	0.0244
P8	0.0389	0.0382	0.0335	0.0369	F4	0.024	0.026	0.0216	0.0239
O1	0.0333	0.0334	0.0385	0.0351	PO4	0.0218	0.0211	0.0256	0.0228
Oz	0.0463	0.0299	0.0285	0.0349	FC2	0.0209	0.0182	0.0166	0.0186
F8	0.0349	0.034	0.0324	0.0338	CP1	0.0159	0.02	0.0188	0.0182
AF3	0.0281	0.0303	0.0377	0.032	P4	0.0154	0.0157	0.0178	0.0163
Fp2	0.0263	0.0417	0.0262	0.0314	Cz	0.0145	0.0184	0.0153	0.0161
AF4	0.0305	0.0255	0.0342	0.0301	CP2	0.0131	0.0135	0.0183	0.015
F3	0.0299	0.0292	0.0286	0.0292	Fz	0.0125	0.0134	0.0129	0.0129

GIs from this table were used in order to create the topoplot at Figure 6.

Next important channels are FC5 (0.0434), O2 (0.0432), P7 (0.0388), and FC6 (0.0387). Which are from emotion component regions such as the middle left and right hemispheres, as well as frontal and parietal lobes (Wang et al., 2019). Other authors have also used these channels, such as F7, F8, and T7–FC2 (Javidan et al., 2021); FP1, T7 and T8 on γ, FC6 on β (Guo et al., 2022); O2, T8, FC5, and P7 (Dura and Wosiak, 2021); FP1–F7 (Taran and Bajaj, 2019); F7, FC5, FC6, O2, and P7 (Wang et al., 2019).

**Figure 7:**
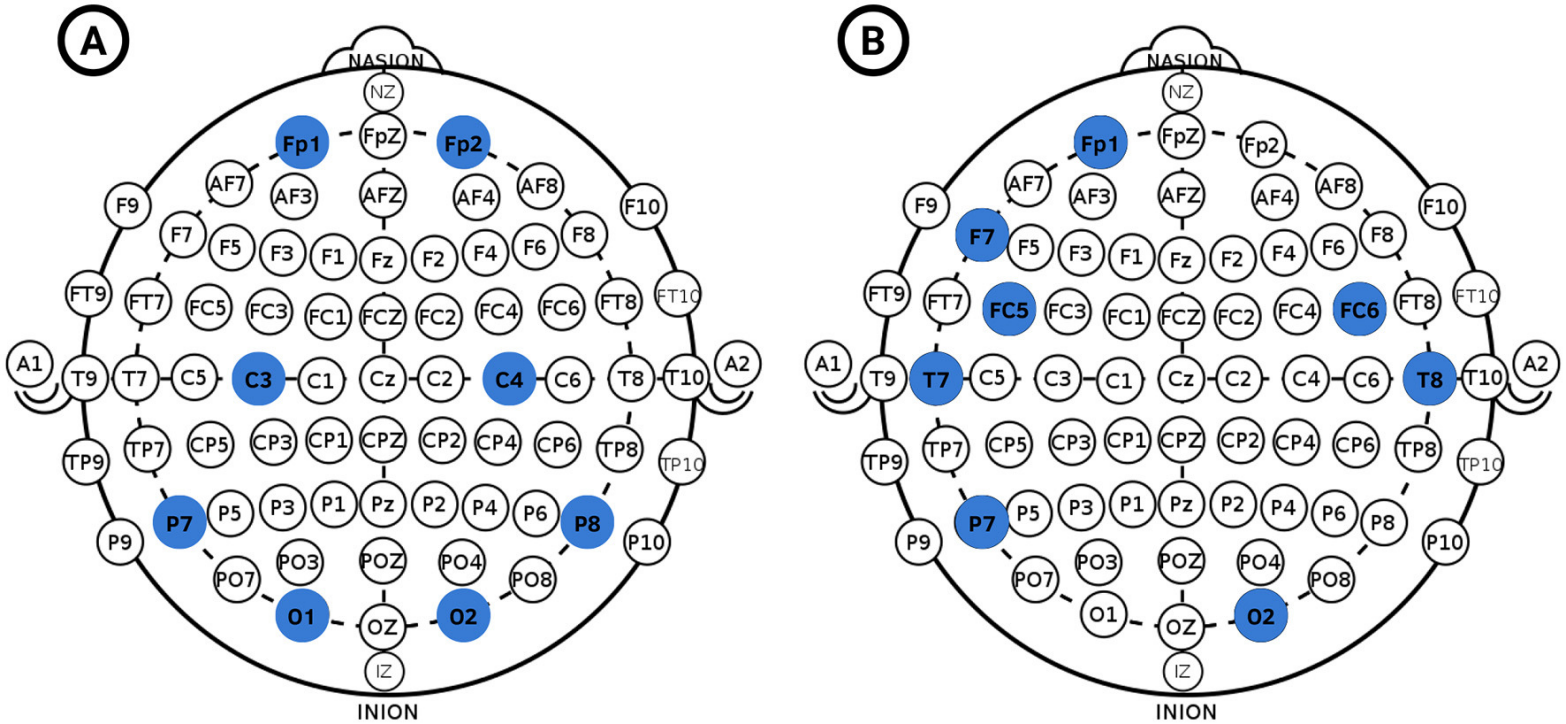
OpenBCI channel configurations, **(A)** Default configuration (FP1, FP2, C3, C4, P7, P8, O1, O2), **(B)** Optimal channel configuration for emotion recognition (Fp1, F7, FC5, FC6, T7, T8, P7, O2).

In this sense, the proposed set of eight channels consists of four frontal channels (Fp1, F7, FC5, FC6), two temporal channels (T7, T8), one parietal channel (P7), and one occipital channel (O2); which correspond to five channels from the left hemisphere and three channels from the right hemisphere, and not including any channels from the previously established central and central-parietal lobes. These results suggest that this set of eight EEG channels would allow us to obtain an optimized version of the model in future iterations. Since the OpenBCI system allows channel reconfiguration, it could be easily implemented to measure EEG signals from such electrodes. So instead of the default OpenBCI channel configuration shown in Figure 7A), the proposed set of channels would be used as in Figure 7B).

### 4.3 Model selection

**Figure 8:**
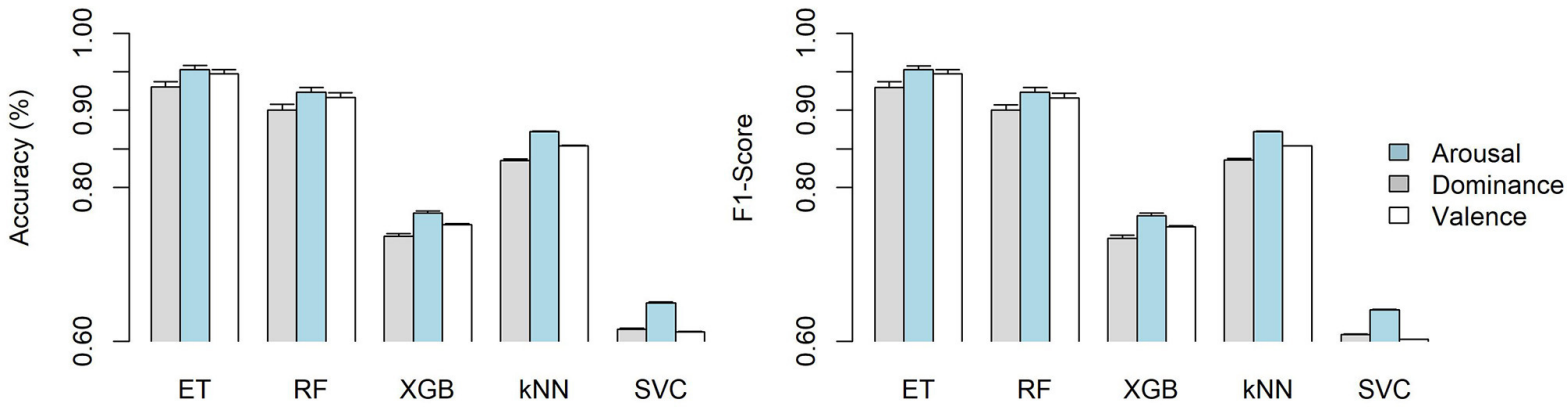
VAD classification models evaluation comparison on Accuracy and F1-score using the best features obtained from the channel selection process, averaged across eight-folds using shift-based cross-validation.

After the channel selection analysis described in Section 4.2 was performed, five different classifier models were trained using these eight channels and their respective PSDs; their results on both accuracy and F1-score are shown in Figure 8. The Extra-Trees model achieves the best accuracy and F1-score, as it performed better than the rest of the models ($\bar{x}=94.35\%$ accuracy, $\bar{x}=94.34$ F1-score) on all VAD components, with only the Random Forest model following behind. Even though RF performed similarly to ET, it performed slightly worse ($\bar{x}=91.35\%$ accuracy, $\bar{x}=91.32$ F1-score) on all VAD components separately; thus, ET was selected as it got closer to 95% on both accuracy and F1-score. In order to avoid doing an expensive computation, these models were trained using an extract of the training data, taking into account every 16 steps; this led to a reduction in the time needed for model selection.

In constructing the Extra-Trees model, decision trees are generated from the entire dataset. Unlike traditional tree algorithms where the best split among all possible splits is chosen, in the Extra Trees methodology, splits are randomly selected for each candidate feature, and the best of these randomly generated splits is used. When combined with ensemble techniques where multiple trees are built and averaged, this randomness often produces a model that is less prone to overfitting. Additionally, this random selection eliminates the need for bootstrapping in sampling, meaning the whole sample is used in constructing each tree (Géron, 2019).

### 4.4 Feature selection

In order to determine the optimal number of features, the Extra-Trees' GI was applied on all the 8-channel's PSD and index features. Furthermore, a series of Extra-Trees models were fitted using the best 25–35 features. Results of the accuracy of scores on Valence, Arousal, and Dominance prediction with different numbers of features with these models are shown in Table 5. Highlighted results show the best number of features for each label and the best overall average. The average accuracy of the best number of features for each channel was calculated; there can be a decrease in average accuracy using the best 34 features than the best 35 features, so 34 features were selected as the optimal number of features to be used in the final model. In this sense, a final Extra-Trees model was fitted using the best 34 features for each VAD component.

**Table 5:** Accuracy comparison of VAD predictive models using different number of features.

**Number of features**	**Valence**	**Arousal**	**Dominance**	**Average**
25	0.945	0.925	0.948	0.939
26	0.945	0.925	0.948	0.939
27	0.946	0.927	0.950	0.940
28	0.947	0.927	0.950	0.941
29	0.947	0.930	0.951	0.942
30	0.947	0.933	**0.951**	0.943
31	0.948	0.933	0.951	0.944
32	0.948	**0.935**	0.951	0.944
33	**0.949**	0.935	0.952	0.945
34	**0.949**	0.936	**0.952**	**0.946**
35	0.949	**0.936**	0.952	0.946

Bold values represent the best performance for each model.

Table 6 shows the final chosen features for each of the VAD-trained models. There are a total of 34 features for each VAD classification model. Powerbands are ordered according to their most prevalent powerband in descending order. It can be seen that in the three models (VAD), the most predominant features were from θ, γ, β, and α, as delta only had 1 feature on Valence and 1 feature on Arousal. Furthermore, θ, β, and γ had the most number of channels, with 23, 24, and 24 channels, respectively, following α with 17 channels. It is quite interesting that the γ bandpower is still prevalent, as shown in the channel selection analysis on Section 4.2, and that the higher frequency features are the most related to emotions, as also suggested in the same section at Table 3. The hybrid feature selection method implemented, which used Pearson's linear correlation coefficient and Extra-Trees' non-linear GI, led to better generalization across the dataset, thus gathering essential insights that lead to optimized performance on 8-channel emotion recognition while including additional index features.

**Table 6:** Top 34 features for Valence, Arousal, and Dominance.

**Value**	**Powerband**	**Channel**
Valence	γ	F7, P7, Fp1, FC5, T8, T7, O2, FC5
	β	Fp1, FC6, T8, F7, T7, P7, O2
	α	FC6, F7, T7, Fp1, FC5, T8, P7
	θ	FC6, F7, Fp1, T8, FC5, T7, P7
	δ	O2
	Engagement	FC5
	Fatigue	P7
	Excitement	P7
Arousal	γ	O2, Fp1, T8, F7, FC5, T7, P7, FC6
	β	FC5, Fp1, T7, F7, P7, FC6, T8, O2
	α	P7, FC6, FC5, T7, F7, T8, Fp1, O2
	θ	FC6, Fp1, F7, T8, P7, T7, FC5, O2
	δ	O2
	Engagement	O2
Dominance	γ	FC6, Fp1, T8, T7, FC5, P7, F7, O2
	β	FC5, T8, T7, Fp1, FC6, P7, F7, O2
	α	FC6, FC5, T8, T7, Fp1
	θ	FC6, F7, P7, FC5, T7, Fp1, T8
	Engagement	FC5, Fp1, FC6
	Fatigue	P7
	Excitement	FC5, FC6

**Figure 9:**
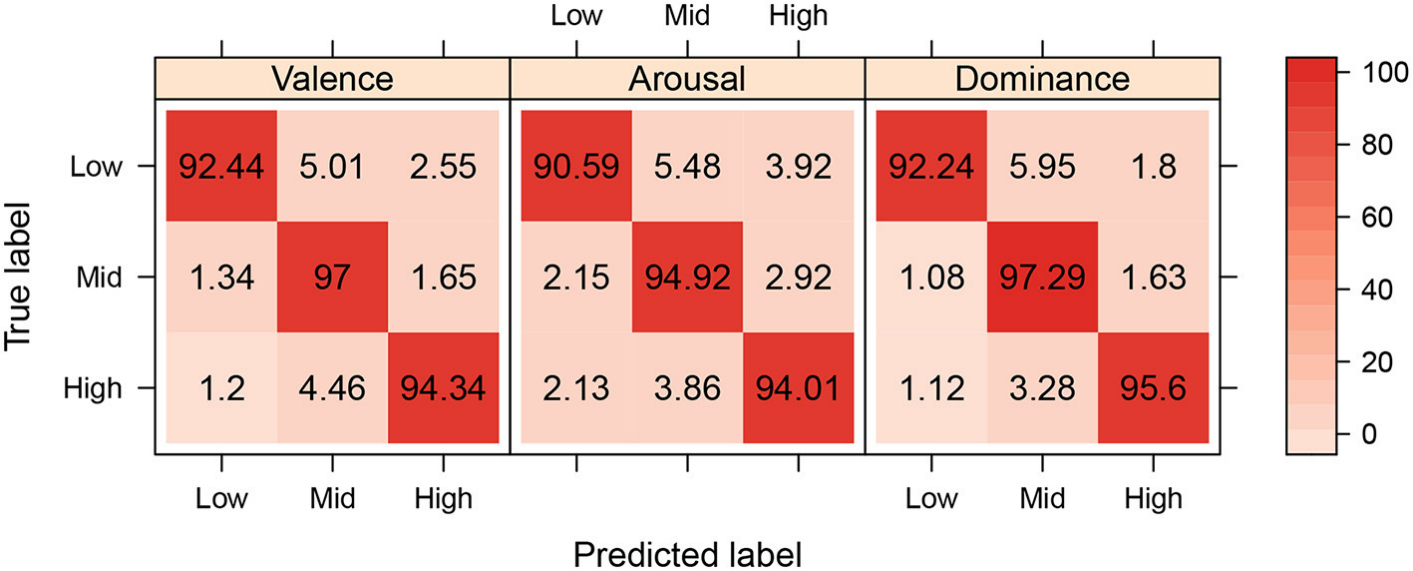
In confusion matrices for each VAD component model, the highest accuracy is expected to be in the diagonal element in each matrix, as it represents a correct classification of a predicted label according to the true label.

The best model's performance (extra-trees with 34 features) achieved an accuracy of 0.946 for Valence, 0.932 for Arousal, and 0.950 for Dominance, with an average accuracy of 0.942. The respective confusion matrices are shown in Figure 9. It can be seen that, overall, the accuracy for each of the quadrants is >0.90, and there is not a clear sign of miss-classification of each of the true and predicted labels, thus showing that residuals are randomly distributed and that high accuracy is balanced among classes of each of the models. However, there seems to be low prediction power when predicting low label on each of the VAD components, and it appears to be increasing at the high label, with peaking accuracy at the mid label, which might be due to human bias when selecting their VAD component level, due to generally pick the middle number thus stating average emotion.

## 5 Conclusions

After all previous analyses were performed, a final model was trained. The chosen model was an Extremely Randomized Trees Classifier (Extra-Trees), as it showed significantly higher precision than the rest of the chosen models. The final features are shown in Table 6, calculated with 5-second time windows, using the proposed channels for the optimal OpenBCI Ultracortex IV configuration at Figure 7B).

The current model is based on PSD estimations only. However, different types of features could add higher complexity, such as neural connectivity metrics. Functional connectivity metrics could be added to the model, for instance, between each channel opposite pair FC5-FC6, T7-T8, as well as other types of bipolar connections such as FP1–F7 (Taran and Bajaj, 2019), in addition to T7-P7 and T7-FP1 (Meyers et al., 2021), that have been reported previously as efficient neural markers.

It is also important to note that the model can be expanded to include variables other than EEG. Integrating other physiological measures, such as Electro-oculography (EOG), Electromyography (EMG), and Electrocardiography (ECG), can bolster the robustness and accuracy of our emotion prediction. These complementary measures provide a multifaceted view of the human emotional response, ensuring a more comprehensive analysis (Koelstra et al., 2012). A future iteration of this algorithm will include such variables, into account, aiming to increase the complexity and accuracy of the model.

The study presents certain limitations that need to be addressed in the near future. Firstly, the choice of the OpenBCI Ultracortex Mark IV for the real-time application, which only contains eight available channels for data collection, presents constraints on the granularity and amount of data that can be captured. It would be valuable to explore options such as incorporating a Daisy extension to expand the helmet to 16 channels or exploring alternative EEG devices with larger electrode arrays to extend these data acquisition capabilities further.

Moreover, relying on a dataset derived from a relatively small sample size of 32 participants is another limitation worth considering. While this sample size was sufficient for this study, it may not adequately represent a broader population. This limitation could impact the generalizability of the overall model on a more comprehensive range of individuals and scenarios. Future researchers may benefit from including a more diverse and extensive dataset to mitigate this limitation to ensure robustness in the model's generalization capabilities.

Future steps of this work will also include the implementation of the real-time algorithm into experimental tests of the users while interacting with the immersive platform, and the generated emotions will be compared to the ones experienced by users in a (non-immersive) control group. Moreover, the inclusion of more biometric signals will offer more insights into the interactions experienced by test subjects when presented with these different scenarios.

Despite the technical limitations this work faced, it is important to remark the high accuracy of the models, as well as the real-time nature of the proposed framework, which opens a window for a broad repertoire of applications. Given that the creation of this algorithm will be applied into the context of NeuroHumanities, several implementations can be explored in the future. One application of our work is the development of assistance systems for educators, that provide a behavioral evaluation of emotional components in different teaching approaches and study plans (Bachler et al., 2023). Another application which can be explored with this work is in the NeuroArts field (Cebral-Loureda et al., 2023); for instance, an exploration of how the brains (of artists and audiences) react during the recreation of specific emotions while performing, and an evaluation of the effectiveness in the process of conveying emotions. A third possible implementation is in the (recently in boom) field of Human Fluorishing (Manuel Cebral-Loureda, 2022). In this sense, the proposed framework can be used to look for strategies that promote the appearance of positive emotions in different types of activities (e.g., wellness, daily life, work-related).

Only three applications were exemplified previously, however, the exploration of emotional components can also be implemented in the fields of Entertainment, Healthcare, Industry, Marketing, among others, in order to assess how different users (emotionally) react to services or products, and the evaluation of the obtained results might be used to provide better solutions and to generate more positive experiences to the final users.

## Data availability statement

The original contributions presented in the study are included in the article/supplementary material, further inquiries can be directed to the corresponding author.

## Ethics statement

The studies involving humans were approved by Queen Mary University London, United Kingdom. The studies were conducted in accordance with the local legislation and institutional requirements. Written informed consent for participation was not required from the participants or the participants' legal guardians/next of kin in accordance with the national legislation and institutional requirements.

## Author contributions

MB-R: Writing – original draft, Writing – review & editing, Data curation, Formal analysis, Investigation, Methodology, Software, Validation, Visualization. MOC-L: Data curation, Formal analysis, Investigation, Methodology, Software, Validation, Visualization, Writing – original draft, Writing – review & editing. CO-R: Investigation, Methodology, Visualization, Writing – original draft. PR-S: Investigation, Methodology, Writing – original draft. MC-L: Conceptualization, Funding acquisition, Resources, Validation, Writing – review & editing. CV-S: Conceptualization, Formal analysis, Investigation, Project administration, Supervision, Writing – review & editing. JL-S: Funding acquisition, Project administration, Supervision, Writing – review & editing. MR-M: Conceptualization, Funding acquisition, Investigation, Methodology, Project administration, Resources, Supervision, Visualization, Writing – original draft, Writing – review & editing.
